# TBF-YOLOv8n: A Lightweight Tea Bud Detection Model Based on YOLOv8n Improvements

**DOI:** 10.3390/s25020547

**Published:** 2025-01-18

**Authors:** Wenhui Fang, Weizhen Chen

**Affiliations:** School of Electrical and Electronic Engineering, Wuhan Polytechnic University, Wuhan 430048, China; f1602085219@163.com

**Keywords:** tea buds, intelligence, YOLOv8n, distributed shift convolution, computer vision

## Abstract

Tea bud localization detection not only ensures tea quality, improves picking efficiency, and advances intelligent harvesting, but also fosters tea industry upgrades and enhances economic benefits. To solve the problem of the high computational complexity of deep learning detection models, we developed the Tea Bud DSCF-YOLOv8n (TBF-YOLOv8n)lightweight detection model. Improvement of the Cross Stage Partial Bottleneck Module with Two Convolutions(C2f) module via efficient Distributed Shift Convolution (DSConv) yields the C2f module with DSConv(DSCf)module, which reduces the model’s size. Additionally, the coordinate attention (CA) mechanism is incorporated to mitigate interference from irrelevant factors, thereby improving mean accuracy. Furthermore, the SIOU_Loss (SCYLLA-IOU_Loss) function and the Dynamic Sample(DySample)up-sampling operator are implemented to accelerate convergence and enhance both average precision and detection accuracy. The experimental results show that compared to the YOLOv8n model, the TBF-YOLOv8n model has a 3.7% increase in accuracy, a 1.1% increase in average accuracy, a 44.4% reduction in gigabit floating point operations (GFLOPs), and a 13.4% reduction in the total number of parameters included in the model. In comparison experiments with a variety of lightweight detection models, the TBF-YOLOv8n still performs well in terms of detection accuracy while remaining more lightweight. In conclusion, the TBF-YOLOv8n model achieves a commendable balance between efficiency and precision, offering valuable insights for advancing intelligent tea bud harvesting technologies.

## 1. Introduction

The tea tree stands as a pivotal agroforestry cash crop globally, with approximately 32% of countries and regions cultivating tea plantations. The scale of this cultivation is witnessing significant expansion. Tea leaves and buds are processed into diverse products, making them one of the world’s most popular beverages [1]. Notably, global tea consumption has surged at an annual rate of 4.5% over the past decade. As of 2023, China’s tea gardens have expanded to cover approximately 7,788,245 acres, reflecting a year-on-year increase of 3.09%. Simultaneously, China’s total tea production has reached 3,339,500 tons, marking an annual growth of about 4.98%. Despite advancements in agricultural science and tea cultivation techniques, the tea harvesting process faces considerable challenges. Given the high economic and nutritional value of tea buds, current domestic harvesting is predominantly manual. This method suffers from substantial drawbacks, including high costs, slow harvesting speed, and increased labor intensity. Moreover, the time-sensitive nature of tea bud harvesting, coupled with a projected future increase in production, exacerbates labor shortages and results in delays that adversely affect economic efficiency.

With the rapid development of artificial intelligence, more and more efficient tea-testing technologies are emerging. These technologies have made tea bud picking more accurate, and artificial intelligence technology is driving advancements in the tea industry, bringing to it more reliable and accurate technology and improving its quality as a whole [2]. Tea-picking robots deployed with tea bud detection technology can accurately identify and locate tea shoots and leaves that meet tea quality standards, thus bringing intelligence and accuracy to the tea industry. Due to the introduction of intelligent mechanical equipment, picking efficiency has been significantly improved, labor costs have been reduced, and the sustainable development of the quality tea industry has been supported [3]. Consequently, the mechanization and intelligent automation of tea bud harvesting has become imperative, necessitating the effective localization, detection, and classification of tea leaves. To address these challenges, we propose the TBF-YOLOv8n, a lightweight deep-learning model specifically designed for tea bud detection. Recognizing the limited computational power of embedded devices and the high computational demands of conventional deep learning models, this study aims to develop an efficient solution for intelligent tea bud harvesting. In recent years, the integration of intelligent technologies, such as image processing and deep learning, has markedly advanced the field of modern agriculture. Notable breakthroughs have emerged in various domains, including pest and disease detection, fruit identification, and yield prediction. For instance, Tian et al. [4] acquired images of apples at different growth stages and employed image enhancement techniques alongside the DenseNet method to process lower-resolution feature maps within the YOLOv3 framework. This integration notably improved network performance, leading to the effective detection of apples using the proposed YOLOv3-dense model. Similarly, Liu et al. [5] optimized the feature layers of YOLOv3 through the implementation of an image pyramid, enabling the rapid and accurate detection of tomato pests and diseases, thereby providing a critical reference for intelligent pest management. Gai et al. [6] replaced the original CSPDarknet53 backbone in YOLOv4 with DenseNet, utilizing a Leaky ReLU loss function, resulting in a 0.15 increase in average accuracy for cherry fruit detection. Wang et al. [7] counted maize stands in videos captured by unmanned aerial vehicles (UAVs) using YOLOv3 and the Kalman filter counting with 98% accuracy for online maize counting. Li et al. [8] further enhanced the YOLOv8s model by incorporating GhostNet and Triplet Attention and introducing the ECIOU loss function, yielding a 50.2% reduction in model size while improving average accuracy by 0.2% for maize disease detection. Jia et al. [9] adapted YOLOv7, integrating MobileNetV3 to minimize parameters, and employed coordinate attention (CA) and SIOU metrics, achieving 92% accuracy in rice pest and disease detection, with an average accuracy of 93.7%. Lastly, Xu et al. [10] proposed the YOLO-RFEW (RFA Conv, EMA, WIOU) model for melon ripeness detection, utilizing RFAConv in place of traditional convolution layers and incorporating FasterNet and EMA attention mechanisms to enhance C2f, resulting in an accuracy of 93.16%, all while maintaining a lightweight and efficient performance profile. These advancements underscore the transformative potential of deep learning techniques in enhancing agricultural productivity and sustainability.

The detection of tea buds presents unique challenges due to their small size, mixed backgrounds, and high density, which contrast with other crop detection targets that are more distinct from their surroundings. These challenges necessitate the development of lightweight models suitable for deployment on tea-picking robots. Recent advancements in tea-related fields have included work on bud detection and localization [11], pest and disease identification [12], and the classification of tea types [13]. Various methodologies have been proposed for tea bud recognition, encompassing traditional machine learning, deep learning, and transfer learning approaches. Due to the varied environment of tea bud harvesting and the fact that tea buds grow in an environment that is easily obscured by foliage, Li et al. [14] proposed a reliable algorithm based on red, green, and blue depth camera images, which were employed for training on the YOLOv3 model, and the final tea bud accuracy rate reached 93.1%. Yang JW et al. [15] proposed a visual localization method for tea picking point based on RGB-D information fusion, constructed a T-YOLOv8n model for identifying and segmenting the key information of tea, and combined the model with a far and near hierarchical visual tetrafluoro strategy to achieve accurate localization of the picking point—the success rate of their picking point localization reached 86.4%. Xu et al. [16] proposed a detection and classification method using a two-level fusion network with variable universe from different angles of tea picking, which is divided into side and top views. This method combines the advantages of YOLOv3 and DenseNet201 to improve detection accuracy: the detection rate of the side view image reached 95.71%, which is 10.60% higher than that of the top view, providing a theoretical and practical solution to the problem of tea bud detection. This provides both theoretical and practical solutions for solving the complex background of tea bud detection. Cao et al. [17] developed a lightweight detection model by integrating GhostNet and YOLOv5. The GhostNet module was used instead of the CSP module to improve detection efficiency, and EIOU was used as the optimized loss function with an accuracy of 76.31%. Chen et al. [18] designed a tea system in which the detection algorithm combines YOLOv3, semantic segmentation algorithm, and minimum bounding rectangle, as well as designed a picking device coordinated and controlled by a computer and a microcontroller, proposed a visual model for tea leaf classification based on Openmv smart camera, and analyzed the effects of different angles on tea leaf picking. The success rate of bud picking in their study reached 80%, which advances the development of precision agriculture. The pursuit of higher accuracy in tea bud detection will also increase the number of parameters and computation requirements of the model, so in order for tea bud detection to be better deployed on devices with limited computational resources, the model should be kept lightweight to make the model more efficient. Yan et al. [19] combined DeepLabV3 and MobileNetV2 for segmenting and localizing tea leaves, achieving accuracies of 82.52%, 90.07%, and 84.78% for various configurations. Subsequent advancements include enhancements to the Mask Region-based Convolutional Neural Networks(R-CNN)model by Cheng et al. [20], who achieved 86.6% accuracy through new anchors and a revised loss function. Gui et al. [21] utilized lightweight convolution methods, achieving a 9.66% increase in average precision by incorporating BAM attention mechanisms and a new loss function in YOLOv5. Li et al. [22] replaced the YOLOv4 backbone with GhostNet, integrating depth-separable convolutions and attention mechanisms to yield a 1.08% increase in average accuracy while drastically reducing computational complexity. Meng et al. [23] combined multiple advanced techniques within the YOLOv7 architecture, achieving a mean average precision (mAP) of 96.7% and real-time detection capabilities. Furthermore, S. Xie and H. Sun [24] introduced the Tea-YOLOv8s model, enhancing the C2f module with deformable convolution and global attention mechanisms, achieving an average accuracy of 88.27%. Based on the YOLOv5 model, Li HF et al. [25] combined the efficient Simplified Spatial Pyramid Pooling Fast (SimSPPf), the Bidirectional Feature Pyramid Network structure (BiFPN), and employment of the Omni-Dimensional Dynamic convolution (ODConv) to enhance the accuracy of tea leaf detection in complex environments. Gui et al. [26] improved YOLOX through the incorporation of a global context mechanism and enhanced attention modules, achieving a mAP value of 92.71% while significantly reducing computational demands.

In the context of tea bud localization detection, it is imperative to not only pursue high detection accuracy but also to consider the model size and detection speed. This paper presents an efficient detection model for tea buds, termed TBF-YOLOv8n. By incorporating the distributed shift convolution (DSCf) module, which demonstrates superior efficiency compared to the traditional C2f module, the computational load of the model is significantly reduced. Additionally, a lightweight coordinated attention mechanism and dynamic upsampling techniques are integrated to enhance performance. The model effectively achieves a balance between size and speed while ensuring detection accuracy.

The contributions of this study are as follows:

1. A lightweight YOLOv8n-based detection model (TBF-YOLOv8n) is developed specifically for tea bud detection. TBF-YOLOv8n maintains high detection accuracy in small target scenarios while exhibiting low computational complexity.
2. A novel feature extraction and fusion module (DSCf) is proposed, utilizing distributed shift convolution to lower computational complexity while preserving accuracy. The incorporation of a coordinated attention mechanism enables the model to focus on critical regions of interest, and the DySample technique enhances feature recovery, thereby improving detection accuracy.
3. Extensive comparative experiments were conducted on the dataset, demonstrating that TBF-YOLOv8n achieves higher detection accuracy relative to four other models, all while maintaining low computational complexity.

## 2. Methods

### 2.1. The Model Structure of the YOLOv8 Network

With the ongoing advancements in artificial intelligence (AI) technologies, an increasing number of agricultural applications now rely on intelligent systems, particularly in the realm of target detection. Notable algorithms have emerged in the field of agricultural target detection, including the R-CNN series, Single Shot MultiBox Detector(SSD) series, and YOLO series models. Each of these models exhibits distinct advantages and limitations. The R-CNN series is recognized for its high accuracy in the detection of small targets; however, it is notable for its substantial computational resource requirements, which may hinder its applicability in real-time scenarios. Conversely, the SSD series strikes a more favorable balance between accuracy and speed, yet its efficacy in detecting small targets remains suboptimal. In light of these differential strengths and weaknesses, the selection of an appropriate model must consider the specific requirements of the agricultural application at hand, including the size of the targets, the need for real-time processing, and the computational resources available. Thus, ongoing research and development in this area are crucial for optimizing target detection algorithms tailored to the nuanced demands of agricultural contexts.

**Figure 1:**
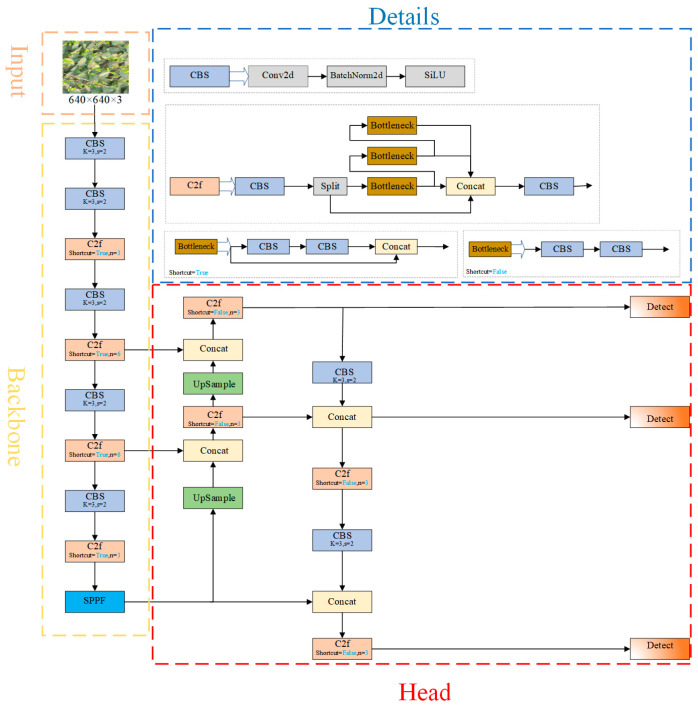
YOLOv8 framework diagram.

YOLO (You Only Look Once) has emerged as a prominent real-time detection model known for its rapid detection capabilities, low complexity, and minimal computational requirements. Among its iterations, YOLOv8 represents a significant advancement. The architecture of YOLOv8 comprises five principal components: the input layer, backbone network, neck network, detection head, and loss function. In YOLOv8, the Efficient Layer Aggregation Networks(ELAN)concept from YOLOv7 is integrated into both the backbone and neck networks, enhancing feature extraction [27]. Additionally, the CSP Bottleneck with 3 convolutions(C3)structure employed in YOLOv5 [28] is substituted with the C2f module, which provides richer gradient representations. The detection head utilizes a decoupled head architecture, enabling improved performance in object localization and classification. Furthermore, the data augmentation strategy incorporates the Mosaic enhancement technique introduced in YOLOX [29], implemented during the final 10 training epochs. These innovations collectively contribute to YOLOv8’s enhanced accuracy and efficiency, reinforcing its position as a leading model in the paradigm of real-time object detection. The YOLOv8 structure is shown in Figure 1.

### 2.2. Improved YOLOv8 Structural Design (TBF-YOLOv8n)

TBF-YOLOv8n is an efficient and lightweight model designed for tea bud detection, built upon an enhanced YOLOv8n framework. The primary contributions of this work are detailed as follows:

Data Enhancement: The model’s generalization ability and robustness were significantly improved through various data augmentation techniques, including random rotation and luminance transformation. These operations promote greater variability in the training dataset, enhancing the model’s overall performance.

Module modification: The conventional C2f module has been replaced by the DSCf (modified C2f module) module. This modification introduces the innovative DSConv in the C2f module, which greatly improves computational efficiency by employing a variable quantization kernel in the distribution offset operation. This not only simplifies the model but also reduces the computational load.

Incorporation of Coordinate Attention: The integration of Coordinate Attention (CA) within the backbone network facilitates the fusion of distant dependencies and spatial information across multiple directions. This enables the model to concentrate on critical regions of interest, thereby enhancing both its robustness and accuracy in the challenging context of tea bud detection.

Dynamic Upsampling Using DySample: The introduction of the DySample dynamic upsampling operator, conceptualized from a point-sampling perspective, optimizes the upsampling functionality while preserving the lightweight nature of the tea bud detection model. This innovation significantly improves computational efficiency during the upsampling process.

Loss Function Improvement: Replacing the previous ‘Completely Intersecting Unions’ (CIOU) with ‘SIOU’, the penalty metrics are redefined to place more emphasis on the angular transformation of the predicted bounding box relative to the ground truth bounding box. This improvement speeds up the convergence process and further improves the model’s performance.

Overall, TBF-YOLOv8n provides a reference for the field of lightweight detection of tea buds, achieving a perfect combination of efficiency and effectiveness through strategic modifications of the architecture and computational techniques.

**Figure 2:**
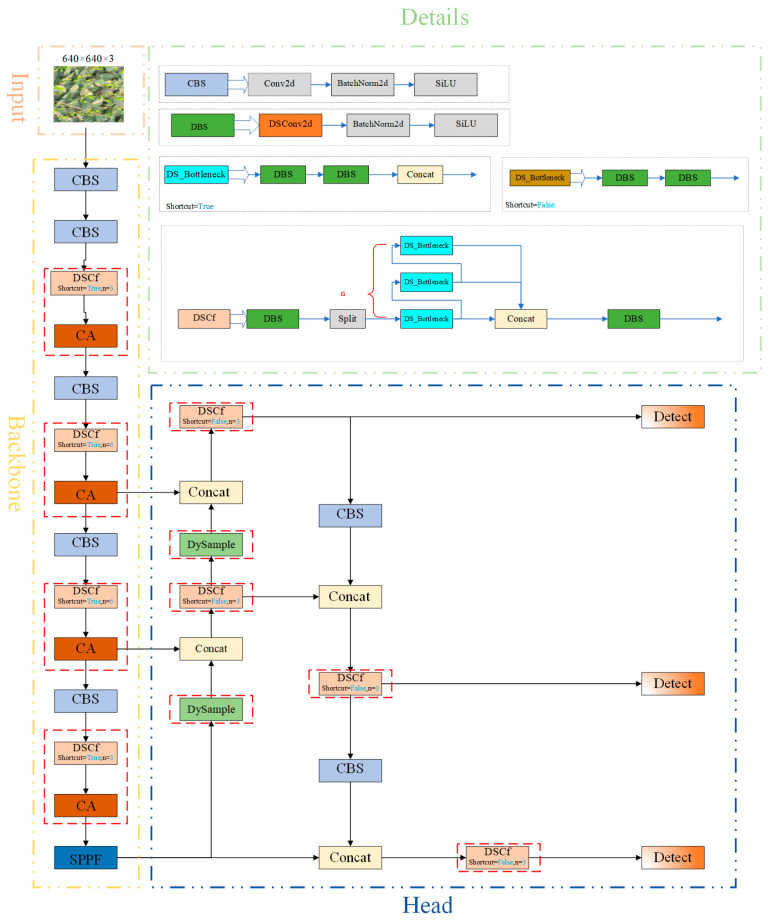
Structure of the TBF-YOLOv8n model. The red dashed box shows the improvement.

This paper describes the structure of the TBF-YOLOv8n model. The traditional C2f module has been replaced by the DSCf module. The DSCf structure retains the principles of the Cross-Stage Partial (CSP) network and consists of two Convolution-Batch Normalisation-Back (CBS) modules and a series of n bottleneck modules. An important innovation of this structure is the replacement of the original convolutional layers with Distributed Shift Convolution (DSConv), which significantly reduces the computational complexity of the model. In addition, a coordinated attention (CA) mechanism is also integrated after the DSCf module, which enhances the feature extraction capability of the model for tea buds while maintaining a lightweight framework and helps to improve detection accuracy. In addition, the upsampling module at the neck of the network has been replaced by the DySample operator. This modification effectively enhances the upsampling functionality without adding additional computational burden. The structural configuration of the TBF-YOLOv8n model is shown in Figure 2, which highlights the improvements in feature extraction and computational efficiency.

#### 2.2.1. Distribution Shifting Convolution

**Figure 3:**
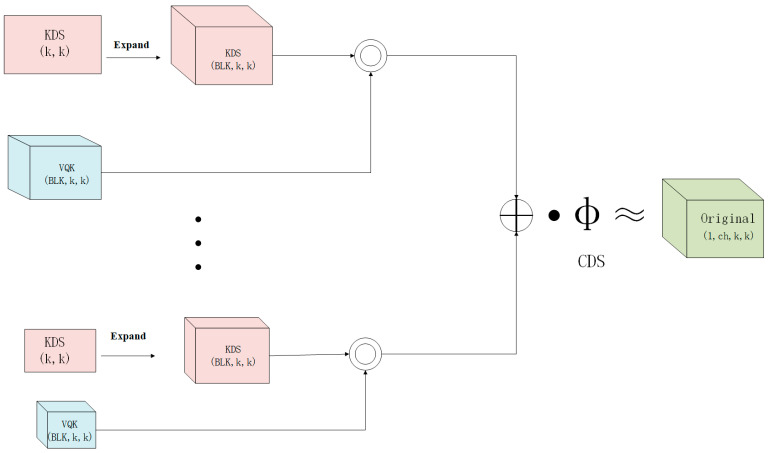
Structure of DSConv. KDS is the kernel distribution shift; CDS is the channel distribution shift; ◎ is the Hadamard operation; the CDS is ϕ; Original represents the original tensor; k is the width and height of the kernel; BLK stands for block size hyperparameter.

In traditional machine learning frameworks, convolutional neural networks (CNNs) have played a pivotal role in target detection applications. Contemporary target detection models, however, incorporate millions of parameters, resulting in substantial computational demands that often prolong processing times and escalate costs, even with the use of Graphics Processing Units (GPUs). Notably, a significant portion of this computational burden arises from the convolutional module, underscoring its critical importance; thus, lightweight architectures have emerged as a key research focus. To enhance computational speed and mitigate the overall complexity of convolutional operations, Nascimento et al. [30] introduced an efficient convolution operator termed Distribution Shifting Convolution (DSConv). DSConv innovatively simulates conventional convolutional operations through two principal mechanisms: quantization and distribution shifting. First, the quantization process employs a Variable Quantized Kernel (VQK), which primarily consists of integer values. This approach facilitates faster convolution operations and minimizes memory usage. Second, the distribution of the VQK is fine-tuned by adjusting the shift components, which effectively simulate the output characteristics of traditional convolution, thereby preserving model performance while significantly reducing computational efforts. These advancements highlight the importance of optimizing convolutional techniques within deep learning to achieve efficient target detection. The DSConv structure is shown in Figure 3.

The quantization process generally takes the number of bits entered in the network as input and uses signed integers for storage, using a 2 s complement [31]. There are the following operations on:

$$w_{q}\in Z|-2^{b-1}\leq w_{q}\leq 2^{b-1}-1$$

where $w_{q}$ denotes the value of each parameter in the tensor.

First, this is achieved by scaling the weights of each convolutional layer with the aim of matching the maximum absolute value of the original weight w with the maximum value after the quantization constraints described above. Afterward, all weights are quantized to their nearest integer. Finally, the new integer-valued weights $w_{q}$ are stored in memory for use in subsequent training and inference.

Distribution shifts were introduced above by adjusting the value of VQK, specifically by kernel distribution shifts (KDS) and channel distribution shifts (CDS). From this point on, the KDS is denoted as ξ, and its offset is denoted as $\xi_{s}$; the CDS is ϕ and its offset is $\phi_{s}$. Their values affect the optimal outcome of the network, so their initialization is very important. The two ways to compute their initialization are by (I) minimizing KL- Divergence, and by (II) minimizing the L2 paradigm. The VQK needs to have a similar distribution to the original weights after the distribution shift, assuming an initial value of $\xi_{s}$. This is accomplished by minimizing the KL- Divergence method described below [30].

(1) $$T_{j}=\frac{e^{w_{j}}}{\sum_{i}e^{w_{i}}},I_{j}=\frac{e^{\xi \cdot w_{qj}}}{\sum_{i}e^{\xi \cdot w_{qi}}}$$

(2) $$\xi =\min\sum_{j}T_{j}\log(\frac{T_{j}}{I_{j}}),\forall (1,\mathrm{BLK},1,1)\mathrm{slice}$$

#### 2.2.2. Coordinate Attention (CA)

Attention mechanisms, inspired by the principles of human visual perception, allow computational models to emulate the human retina’s ability to focus on significant elements within a visual scene [32]. These mechanisms enable models to process data by dynamically allocating computational resources, prioritizing more critical information while filtering out less relevant data. While the incorporation of attention mechanisms generally enhances model accuracy, it often introduces substantial computational overhead. To address these concerns, various lightweight attention mechanisms have been developed, including the Squeeze-and-Excitation (SE) block [33] and the Convolutional Block Attention Module (CBAM) [34]. Early lightweight networks primarily utilized the SE mechanism, which effectively captures channel-wise dependencies but neglects the impact of spatial information. In contrast, the CBAM incorporates convolutional operations to account for positional dependencies alongside channel information. However, the ability of CBAM to capture spatial information is inherently limited to the local vicinity defined by the convolutional kernel, thereby rendering it less effective in modeling long-range positional relationships. In response to these limitations, coordinate attention has emerged as an efficient mechanism capable of integrating extensive positional and channel information [35]. This innovative approach enhances the ability of models to discern and utilize relevant spatial features across broader contexts, thereby improving overall detection performance while mitigating the computational burden associated with traditional attention models.

**Figure 4:**
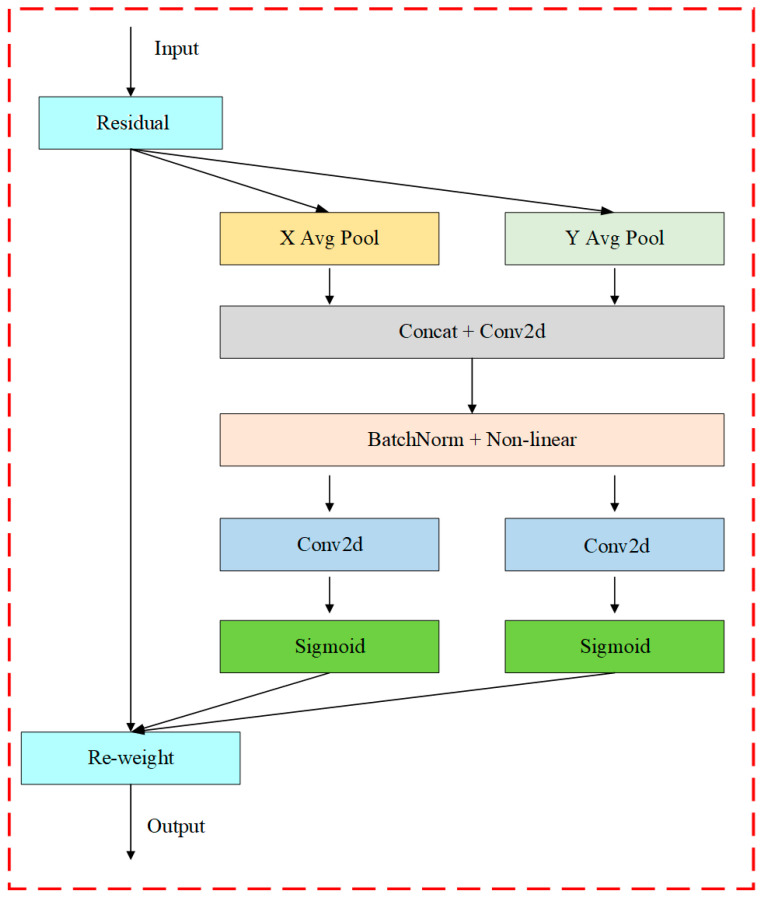
CA structure diagram. “X Avg Pool” and “Y Avg Pool” signify one-dimensional horizontal global average pooling and one-place vertical global average pooling.

The coordinate attention (CA) mechanism enhances the efficacy of channel attention by dividing the feature tensor encoding into two parallel one-dimensional representations corresponding to the x-direction and y-direction. This division serves to mitigate the loss of positional information typically associated with two-dimensional global average pooling, effectively incorporating spatial coordinate information into the encoding process. In this approach, average pooling is simultaneously performed in both the x and y directions, resulting in vertical and horizontal one-dimensional feature tensors. These two tensors are then merged along the spatial dimension, followed by a 1 × 1 convolution operation aimed at reducing the number of channels, thereby decreasing computational complexity. Subsequently, the resultant feature tensor undergoes normalization and processing with a nonlinear activation function. The resulting tensor is then bifurcated into two distinct feature tensors corresponding to the horizontal and vertical dimensions. These tensors are weighted and fused through convolution operations and a Sigmoid activation function, ultimately multiplying by the original input feature map to yield the final output. This methodology not only enhances the model’s ability to capture spatial dependencies but also optimizes computational efficiency, reinforcing the effectiveness of the coordinate attention mechanism in various applications. The CA attention structure is shown in Figure 4.

#### 2.2.3. Dynamic Upsampling Operator (DySample)

**Figure 5:**
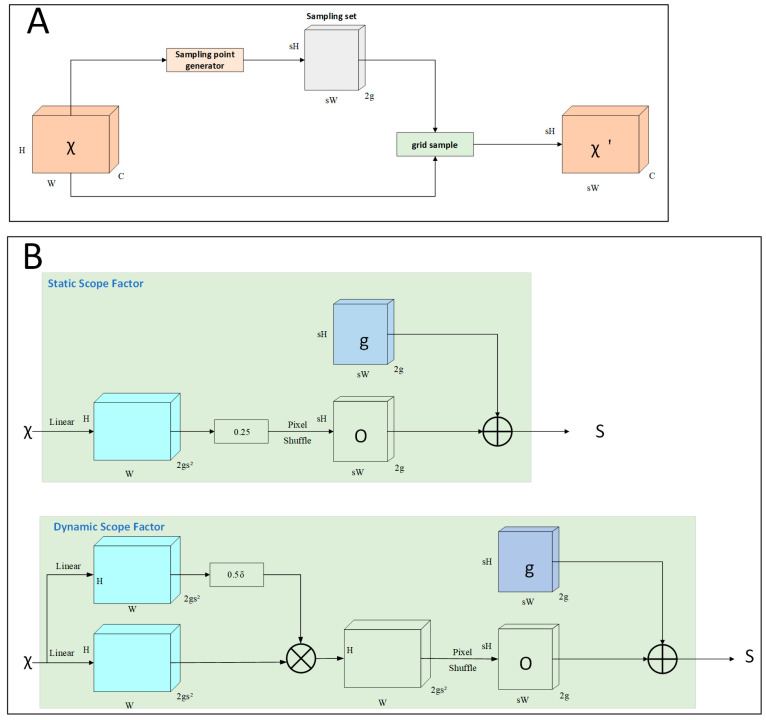
DySample design; χ, O, g, and δ correspond to the input features, upsampled features, offsets, original network, sampling set, and Sigmoid function, respectively. (**A**) The sampling set S is generated by the sampling point generator, and this process is represented by Equation (3); (**B**) denotes the sampling point generator, and the sampling set S consists of the offsets and the original network g Equation (5). There are two different ways of generating the sample set S, which involve two types of offsets: one is static range factor Equation and the other is dynamic range factor Equation (7).

Upon entering the backbone of the neural network, the size of the extracted feature map from the input image is reduced, necessitating subsequent restoration to its original dimensions for further computational processing. This restoration is facilitated by the “upsample” module. Traditional upsampling methods include bilinear interpolation, transposed convolution, and inverse pooling. However, these approaches typically follow a fixed interpolation strategy, encountering limitations in terms of flexibility and efficiency. With the advancement of dynamic networks, several dynamic upsampling techniques have emerged, including CARAFE, FADE, and SAPA. CARAFE [36] employs dynamic convolution for upsampling features, while FADE and SAPA necessitate the availability of high-resolution bootstrap features. Consequently, these dynamic methods often impose substantial computational burdens and require custom CUDA implementations. In the context of lightweight networks, the DySample upsampling operator has been introduced. This innovative method circumvents the computational demands associated with dynamic convolution by utilizing a point sampling approach, thus eliminating the need for specialized CUDA packages. DySample is distinctly advantageous as it can be readily implemented using built-in functions in PyTorch [37], providing a more efficient and accessible solution for upsampling in various applications. The DySample structure is shown in Figure 5.

(3) $$\chi^{\prime }=\text{grid\_sample}(\chi ,S)$$

(4) O=linear(χ)

(5) S=g+O

(6) O=0.25linear(χ)

(7) $$O=0.5\mathrm{sigmoid}({\mathrm{linear}}_{1}(\chi ))\cdot {\mathrm{linear}}_{2}(\chi )$$

Compared with other dynamic upsamplers, DySample does not require high-resolution feature inputs, which reduces the amount of computation required due to dynamic convolution, and does not require a customized CUDA package, which can be accomplished by using the built-in functions of PyTorch, revealing the advantages in terms of the number of parameters, training time, and so on.

#### 2.2.4. SIOU Loss Function

**Figure 6:**
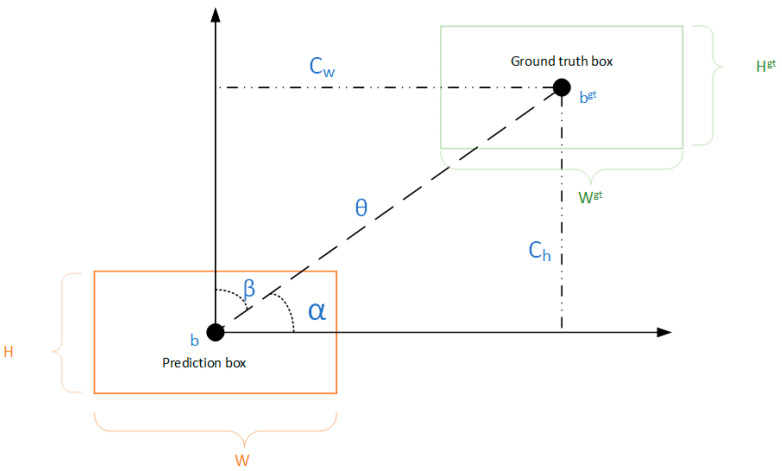
SIOU loss function.

Intersection over Union (IoU) is a critical metric used to evaluate the performance of object detection models, quantifying the degree of overlap between the predicted bounding box and the ground truth box. The SIOU loss function, also known as SCYLLA-IOU, introduces an angle-based penalization metric, which takes into account the angular orientation of the vector between the expected regression and its defined target. This enhancement allows the predicted bounding box to align more rapidly with the coordinate axes, thereby significantly accelerating the convergence process during training [38]. The SIOU loss function comprises four distinct components: Angle Cost, Distance Cost, Shape Cost, and IoU Cost. These components collaboratively contribute to a more nuanced and effective loss calculation, optimizing the model’s ability to achieve precise object localization. A visual representation of the SIOU loss function is provided in Figure 6, illustrating its multifaceted structure and associated costs. The SIOU loss function is shown in Figure 6.

Angle Cost makes the prediction box reach the X or Y axis according to the principle of minimum distance and then approach the Ground truth box along that axis, which can speed up the calculation of the distance between the two boxes (see Equation (8)).

(8) $$\Lambda =1-2\cdot \sin^{2}(\arcsin(\frac{C_{h}}{\theta }-\frac{\pi }{4}))$$

where

(9) $$\theta =\sqrt{{\left(b_{C_{x}}^{gt}-b_{C_{x}}\right)}^{2}+{\left(b_{C_{y}}^{gt}-b_{C_{y}}\right)}^{2}}$$

(10) $$C_{h}=\max(b_{C_{y}}^{gt},b_{C_{y}})-\min(b_{C_{x}}^{gt},b_{C_{x}})$$

$\left(b_{C_{x}},b_{C_{y}}\right)$ and $\left(b_{C_{x}}^{gt},b_{C_{y}}^{gt}\right)$ represent the center of mass coordinates of the prediction box and ground truth box, respectively.

Distance Cost represents the distance between the prediction box and the center of mass of the ground truth box, redesigned based on Angle Cost (see Equation (11)).

(11) $$\Delta =\sum_{t=(x,y)}\left(1-e^{-\rho t\gamma }\right)$$

where

(12) $$\rho_{x}={\left(\frac{b_{C_{x}}^{gt}-b_{C_{x}}}{C_{w}}\right)}^{2}$$

(13) $$\rho_{y}={\left(\frac{b_{C_{y}}^{gt}-b_{C_{y}}}{C_{h}}\right)}^{2}$$

(14) γ=2−Λ

where $C_{w}$ and $C_{h}$ represent the width and height of the minimum outer rectangle of the true bounding box and prediction box, respectively.

The Shape Cost defining equation is shown in Equation (15). Shape Cost takes into account the width-to-height ratio of the true and predicted bounding boxes to make them closer to each other. It represents the level of concern for shape loss control. If it is set to 1, the shape is optimized to reduce the movement between the prediction box, which is generally set in the range of 2 to 6.

(15) $$\Omega =\sum_{t=w,h}{\left(1-e^{-\omega_{t}}\right)}^{\delta }$$

where

(16) $$\omega_{w}=\frac{\left|w,w^{gt}\right|}{\max(w,w^{gt})}$$

(17) $$\omega_{h}=\frac{\left|h,h^{gt}\right|}{\max(h,h^{gt})}$$

where w and h represent the width and height of the prediction box, and W^gt^ and h^gt^ represent the width and height of the ground truth box, respectively.

The basic IOU formula is as follows:

(18) $$\mathrm{IOU}(A,B)=\frac{A\cap B}{A\cup B}$$

where A represents the prediction box, B represents the ground truth box, and IOU represents the overlap rate between them.

In summary, the formula for SIOU is summarized in Equation (19):

(19) $${\mathrm{Loss}}_{\mathrm{SIOU}}=1-\mathrm{IOU}+\frac{\Delta +\Omega }{2}$$

## 3. Material

### 3.1. Data Acquisition and Pre-Processing

**Figure 7:**
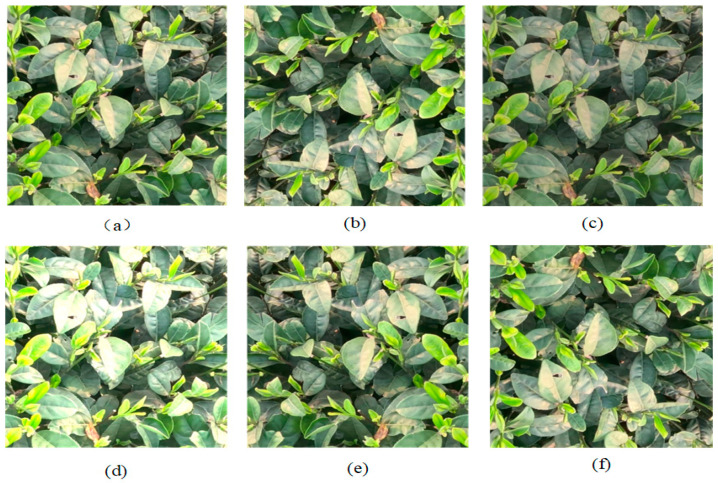
Data preprocessing. (**a**) original image; (**b**) random rotation; (**c**) brightness decrease; (**d**) brightness increase; (**e**) horizontal flip; (**f**) vertical flip.

The dataset utilized in this study is derived from the publicly available TeaBud dataset hosted on the Kaggle platform. It consists of a comprehensive collection divided into three subsets: a training set comprising 5055 images, a validation set containing 562 images, and a test set with 625 images, culminating in 6242 images. The dataset is categorized using one classification and labeled as tea_bud. The photo format is JPG and the image resolution is 676 × 760. To reduce the training complexity, the image resolution is set to 640 × 640 during model training. Tea picking can have multiple complexities, such as light intensity, distance, picking height, and angle. The dataset should be expanded with multiple angles to simulate multiple situations during tea picking so that the model can be better adapted to multiple situations. Therefore, we adopted multiple image processing methods to enhance the data. These techniques include random rotation, brightness adjustment (both darkening and reduction), and horizontal and vertical flipping. The application of these operations significantly increases the diversity of the images, consequently improving the model’s generalization capabilities and robustness. This approach ensures that the model remains adaptable across various environmental conditions, thereby enhancing its overall performance in practical applications. The dataset enhancement is shown in Figure 7.

### 3.2. Experimental Environment

All the experiments in this paper were conducted in the following environments, and the training environments are listed in Table 1 below.

### 3.3. Training Parameters

The values of the parameters applied in the training process are shown in Table 2 below:

### 3.4. Assessment of Indicators

To comprehensively assess the efficacy of the model proposed in this study, several key metrics were selected: Precision (P), Recall (R), Mean Average Precision (mAP), Gigafloating Point Operations (GFLOPs), and the total number of Parameters. Precision quantifies the proportion of positive samples for which the model’s predictions are correct. Specifically, a predicted bounding box is deemed accurate when it sufficiently overlaps with the corresponding ground truth bounding box. This metric provides insight into the model’s ability to avoid false positives, thus contributing to the overall evaluation of its performance. The equation is given below:

(20) $$\mathrm{Precision}=\frac{\mathrm{TP}}{\mathrm{TP}+\mathrm{FP}}$$

Recall indicates the proportion of all true positive samples that the model can find. The equation is as follows:

(21) $$\mathrm{Recall}=\frac{\mathrm{TP}}{\mathrm{TP}+\mathrm{FN}}$$

The average precision is represented by the area under the precision-recall curve (P-R curve) with the following formula:

(22) $$\mathrm{AP}=\int_{0}^{1}P(R)\mathrm{dR}$$

(23) $$\mathrm{mAP}=\frac{\sum_{1}^{N}\int_{0}^{1}P(R)\mathrm{dR}}{N}$$

Since tea bud detection belongs to one classification, N = 1, so AP = mAP.

The assessment of a model’s lightweight nature primarily relies on two key metrics: (I) Gigafloating Point Operations (GFLOPs) and (II) the total number of parameters. These metrics serve to characterize the model’s time complexity and space complexity, respectively. GFLOPs quantify the total number of floating-point operations performed during model training. This metric is commonly employed to gauge the models’ computational efficiency and processing speed. In contrast, the total number of parameters indicates the overall size of the model and the associated storage requirements. This latter metric is crucial for understanding the model’s demand on memory resources during both training and deployment. Together, these metrics provide a comprehensive framework for evaluating the model’s lightweight attributes.

## 4. Experiments and Results

### 4.1. Model Comparison Experiments

#### 4.1.1. Comparison Experiment Between TBF-YOLOv8n and YOLOv8n

**Figure 8:**
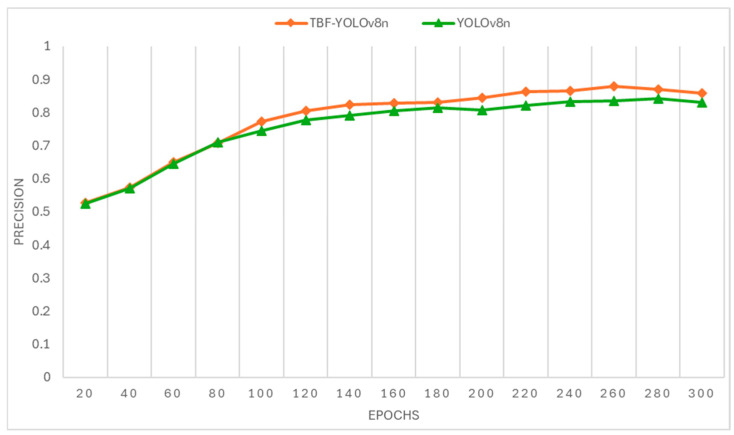
The training process of TBF-YOLOv8n and YOLOv8n.

The dataset used in this study was taken from the Kaggle website using a variety of enhancement techniques. We plotted the training process of TBF-YOLOv8n vs. YOLOv8n as a line graph, the results of which are shown in Figure 8. It can clearly be seen that the training process maintains higher accuracy after 80 epochs. The training is completed after 300 epochs, and the model detection accuracy is finally tested with the validation set. The accuracy of TBF-YOLOv8n is improved by 3.7% over YOLOv8n.

Based on the results of the comparison experiments between TBF-YOLOv8n and YOLOv8n, it can be concluded that the TBF-YOLOv8n precision was improved by 3.7%, that of mAP50 was improved by 1.1%, the number of parameters was reduced by 0.4 M, and the FLOPs were reduced by 3.6 G. Moreover, the recall of the TBF-YOLOv8n has been reduced by 1.2% and the FPS has been reduced by 46.77 frames per second: the results of which are shown in Table 3. As shown in Table 4, the main contribution of TBF-YOLOv8n in reducing the parameters and FLOPs is due to the DSCf module; however, the DySample module and the coordinated attention mechanism have less impact on the parameters and FLOPs. Analyzing by layer, we replaced the 8 C2f modules of YOLOv8n with 8 DSCf modules, and the parameters were reduced by about 0.4 M.

In order to reduce the parameters and FLOPs of the model, we improved the C2f module by replacing the original C2f module with DSCf, which reduces the parameters by 0.4 M and the FLOPs by about 3.6 G, but the consequent effect is a decrease in the detection accuracy; in order to improve the models’ detection accuracy of the tea buds, we added the Coordinated Attention Mechanism, replaced UpSample with DySample, and replaced CIOU with SIOU. These added modules had less effect on the number of parameters and FLOPs, and the FPS was reduced by 46.77 frames per second. In conclusion, TBF-YOLOv8n improves detection accuracy and reduces the number of parameters and FLOPs at the expense of a small amount of FPS and recall, and these sacrifices are justifiable in tea bud detection. The training process of TBF-YOLOv8n and YOLOv8n is shown in Figure 8.

#### 4.1.2. Comparative Experiments with Multiple Models

The results of the comparison experiments are analyzed in two directions: (1) comparison with the lightweight YOLO series base model and (2) comparison with other optimized models. Table 5 summarizes the comparison of the performance metrics, such as precision (P), recall (R), mean age precision (mAP), number of floating-point operations, and total number of parameters of each model. (1) In the comparison of the basic models of the YOLO series, the accuracy of the TBF-YOLOv8n model reaches 87.5%, which is 11.5%, 3.7%, 2.4%, 2.2%, and 2% higher than that of the YOLOv7_tiny, YOLOv8n, YOLOv9_tiny, YOLOv10n, and yolo11n models, respectively. In terms of recall, TBF-YOLOv8n is 5.3% higher than YOLOv9_tiny, but the recall is lower than that of YOLOv7_tiny, YOLOv8n, YOLOv10n, and YOLO11n models, by 2.9%, 1.2%, 2.1%, and 0.7%, respectively. In terms of average precision, TBF-YOLOv8n is higher than the YOLOv7_tiny, YOLOv10n and yolo11n models, and YOLOv7_tiny, YOLOv8n, YOLOv9_tiny, YOLOv10n, and YOLO11n by 3.3%, 1.1%, 6.9%, 0.2% and 0.4%, respectively. In terms of average accuracy, TBF-YOLOv8n significantly reduces the number of floating-point operations compared with the above models by 8.7 G, 3.6 G, 6.2 G, 3.7 G, and 1.7 G, respectively. Finally, in terms of the number of parameters, TBF-YOLOv8n performs the second best, with a reduction of 3.40 M, 0.40 M, 0.1 M, and 0.9 M compared to YOLOv7_tiny, YOLOv8n, YOLOv9_tiny, and YOLOv10n, respectively, and is only 0.3 M higher than YOLO11n. (2) In comparison with other improved models, TBF-YOLOv8n is 0.8% and 3.8% higher than YOLOv5_tea and YOLOv8_tea in terms of precision, and 2.7% and 0.6% lower than YOLOv5_tea and YOLOv8_tea in terms of recall; however, the GFLOPs of TBF-YOLOv8n are 18.2 G and 4.9 G lower than those of YOLOv5_tea and YOLOv8_tea by 18.2 G and 4.9 G, and the number of parameters of the model is reduced by 6.59 M and 2 M, respectively. These findings underscore the enhanced performance of the TBF-YOLOv8n model in terms of both accuracy and computational efficiency.

In conclusion, compared with the best and latest YOLO11n model, although TBF-YOLOv8n is only 0.3 M higher than it in terms of the total number of parameters, TBF-YOLOv8n outperforms YOLO11n in terms of detection accuracy, average accuracy, and FLOPs. Compared with other models optimized for tea bud detection, TBF-YOLOv8n is ahead of YOLOv5_tea in terms of accuracy, FLOPs, and number of parameters, and is only 0.1% lower than YOLOv5_tea in terms of average accuracy. These results indicate that the improvement of the TBF-YOLOv8n model is successful and highlights its lightweight and efficient features in tea bud detection.

### 4.2. Ablation Experiments

#### 4.2.1. TBF-YOLOv8n Ablation Experiment

To evaluate the effectiveness of each component in the model, we designed five sets of ablation experiments, the results for which are shown in Table 6. Initially, replacing the C2f module with the DSCf module resulted in a significant reduction in the number of floating-point operations (FLOPs) by 45.7% and a reduction in the total number of parameters by about 14%, but with a slight decrease in the mean accuracy (mAP) by 0.8%. Based on this, we introduced a coordinated attention (CA) mechanism after the DSCf module in the backbone network. This improvement resulted in a 1% improvement in mAP and a 1.7% improvement in precision (P). Subsequently, we replaced the original loss function with the SIOU loss function, which not only sped up convergence but also improved P by 0.7%. Finally, the use of a lightweight upsampling operator, DySample, further improved the *p*-value by 1.2% and mAP by 0.7%. In conclusion, these experimental results show that the enhanced TBF-YOLOv8n model is lighter and more accurate than its predecessor YOLOv8n. Therefore, the model is suitable for devices with limited hardware resources and provides a valuable reference for advancing mechanized intelligent tea picking.

#### 4.2.2. Loss Function Ablation Experiments

In this section, we describe experiments conducted on the ablation of the loss function, the results of which are shown in Table 7. The experimental results show that SIOU improved precision, mAP50 and mAP50-95, and is 0.5% lower than CIOU in recall.

Comparing the differences between CIOU and SIOU, they both consider factors such as centroid distance and overlap area, but CIOU adds a shape loss term while SIOU introduces an angular loss. The angular loss of SIOU considers the effect of the angle between the prediction box and ground truth box on the bounding box regression, so SIOU can better capture the geometric difference between the prediction box and the ground truth box, thus realizing more accurate bounding box regression. The shape loss of CIOU is the most important factor in the detection of tea buds, and the shape loss of SIOU is the most important factor in the detection of tea buds. Loss is often suitable for dealing with targets in which the shape difference is large. In the process of tea bud detection, tea buds are small in size, high in background complexity, and small in shape difference, so more accurate bounding boxes are needed. Therefore, SIOU is more suitable for tea bud detection than CIOU.

### 4.3. Visual Inspection

**Figure 9:**
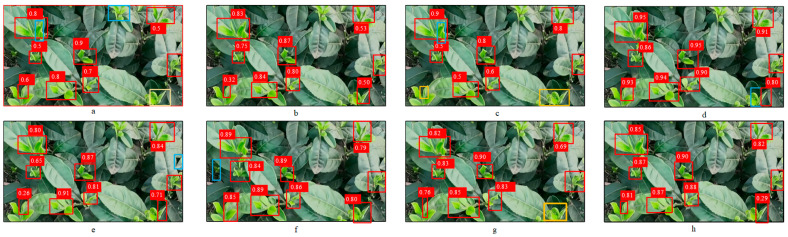
Results of the five model tests. (**a**) YOLOv7-tiny; (**b**) YOLOv8n; (**c**) YOLOv9-tiny; (**d**) YOLOv10n; (**e**) YOLO11; (**f**) YOLOv5_tea; (**g**)YOLOv8_tea; (**h**)TBF-YOLOv8n. Red boxes represent correct detections, blue boxes represent incorrect detections, and yellow boxes represent not detected.

To compare the detection performance between the models more intuitively, the best weights trained by each model were used for testing, and the results are shown in Figure 9:

The results show that there is still great potential for improvement in detecting tea buds, as leakage and misclassification were found in the practical evaluations. Among the models evaluated, TBF-YOLOv8n and YOLOv8n had the lowest number of missed detections and no misdetections; however, TBF-YOLOv8n had a higher average accuracy of detection compared to YOLOv8n. Compared to TBF-YOLOv8n, YOLOv7_tiny had an improved number of missed detections and false detections but a lower overall average accuracy of detection. Of all the models, YOLOv9_tiny had the worst performance, with the highest number of missed detections and a corresponding decrease in detection accuracy, suggesting that it is insufficient for detecting tea buds, especially in complex environments characterized by small targets. YOLOv10n had the highest detection accuracy, but it had a higher false detection rate than TBF-YOLOv8n, and both FLOPs and number of parameters were higher than those of TBF-YOLOv8n. YOLO11n is close to TBF-YOLOv8n in terms of the model size, but it had a detection accuracy lower than that of TBF-YOLOv8n by 5.5%, and its false detection rate was higher. YOLOv5_tea had the second highest average detection accuracy, but all of its model sizes were larger than those of TBF-YOLOv8n and had higher miss detection rates. In conclusion, the TBF-YOLOv8n model not only maintains the lightweight feature but also achieves higher detection performance in tea bud detection, highlighting its applicability to real-world applications in this field. The test results are shown in Table 8.

## 5. Discussion

The TBF-YOLOv8n model combines the DSCf module, dynamic upsampling operator, Coordinate Attention (CA), and SIOU loss function on top of YOLOv8n to enhance tea bud detection. The model significantly reduces computational effort by about 44.4%, reduces the number of parameters by 13.3%, achieves an accuracy of 87.5%, and becomes more lightweight.

However, several limitations and challenges persist. Firstly, the dataset does not account for variations in shooting angles, leading to inconsistencies in image acquisition; it has been documented that images captured from certain angles, such as lateral views, often yield superior detection accuracy [16]. Secondly, despite their varying economic values, the model cannot currently classify different types of tea buds. Intelligent tea bud harvesting systems need to maintain both high detection accuracy and the ability to differentiate between bud categories.

Consequently, future research should focus on the more granular detection of tea buds, such as distinguishing between single buds, “one bud and one leaf”, and “one bud and two leaves”. Additionally, precise segmentation of the tea bud picking area [40], effective prediction of tea yield [41], the design of accurate robotic arm structures for harvesting [3], and the development of an intelligent tea bud detection system are critical areas for exploration. Future investigations must incorporate more effective methodologies aimed at enhancing the model’s generalization capability and improving algorithmic accuracy, which is vital for advancing intelligent tea bud harvesting technologies.

## 6. Conclusions

To facilitate the deployment of the tea bud detection model, we propose the TBF-YOLOv8n lightweight tea bud detection algorithm. The model starts with a more efficient Distributed Shift Convolution (DSConv), which enhances the C2f component by adjusting the distribution of variable quantization kernels, thus significantly reducing the computational requirements and enabling a more lightweight architecture. Subsequently, the introduction of the Coordinate Attention (CA) mechanism allows for the fusion of spatial information across varying orientations and distances, thereby improving the detection accuracy of the model. Moreover, we replace the conventional loss function with the SIOU loss function, enabling the predicted frames to approximate the ground truth with greater rapidity, which in turn accelerates model convergence. Finally, we incorporate the dynamic upsampling operator, DySample, which enhances detection accuracy while preserving the model’s lightweight characteristics through a point-sampling design.

Comparison tests with other models using the same dataset show that compared to the original YOLOv8n model, TBF-YOLOv8n improves accuracy by 3.7%, mean average precision (mAP) by 1.1%, the number of floating-point operations by about 44.4%, and the number of parameters by 13.3%. It is worth noting that the total number of parameters of the TBF-YOLOv8n model is smaller than that of the YOLOv7_tiny, YOLOv8n, YOLOv9_tiny, YOLOv10n, YOLOv5_tea, and YOLOv8_tea models, and is only 0.3 M higher than that of the YOLO11n; however, the TBF-YOLOv8n maintains the highest accuracy.

In conclusion, the TBF-YOLOv8n model serves as a valuable reference for lightweight implementations in smart tea-related applications. Future research will focus on the adaptation of this model for real-time tea bud detection on mobile devices, the development of a robotic arm for tea bud harvesting, and the design of an integrated agricultural management system. In addition, the model can provide insights applicable to other agricultural inspection tasks such as fruit harvesting and pest control, thus effectively informing the development of smart agriculture.

## Figures and Tables

**Table 1:** Experimental configuration and training environment.

Environmental Parameter	Value
CPU	Intel i5 13600KF
GPU	GEFORCE RTX 4060Ti
RAM	16 G × 2
Video memory	16 G
Operating system	Windows11
Deep learning framework	PyTorch
Cuda	11.8
OpenCV	4.10.0

**Table 2:** Experimental configuration and training environment.

Parameter	Value	Parameter	Value
Learning Rate	0.01	Batch Size	32
Image Size	640 × 640	Epoch	300
Momentum	0.937	Weight Decay	0.0005
Optimizer	Auto		

**Table 3:** Results of loss function ablation experiments.

Model	P/%	R/%	${mAP}_{50}/\%$	FLOPs/G	FPS
YOLOv8n	83.8	**75.6**	83.9	8.1	**677.37**
TBF-YOLOv8n	**87.5**	74.4	**8** **5.0**	**4.5**	630.60

**Table 4:** Comparison of parameters of different layers of TBF-YOLOv8n and YOLOv8n. The numbers in parentheses represent the number of modules.

Model	Layer	Parameters	Layer	Parameters
YOLOv8n	C2f(8)	1,517,120	Upsample(2)	0
TBF-YOLOv8n	DSCf(8)	1,097,280	Dysample(2)	12,352
	CA(4)	12,576		

**Table 5:** Individual performance metrics of the model.

Model	P/%	R/%	${mAP}_{50}$/%	FLOPs/G	Params/M
YOLOv7_tiny	76.0	**77.3**	82.0	13.2	6.01
YOLOv8n	83.8	75.6	83.9	8.1	3.01
YOLOv9_tiny	85.1	69.1	78.1	10.7	2.62
YOLOv10n	85.3	76.5	84.8	8.2	2.70
YOLO11n	85.5	75.1	84.6	6.3	2.58
YOLOv5_tea [25]	86.7	77.1	85.1	22.7	9.20
YOLOv8_tea [39]	83.7	75.0	83.6	9.4	4.61
TBF-YOLOv8n	**87.5**	74.4	85.0	**4.5**	**2.61**

**Table 6:** Results of ablation experiments.

Model	P/%	R/%	${mAP}_{50}/\%$	FLOPs/G	Params/M
YOLOv8n	83.8	**75.6**	83.9	8.1	3.01
YOLOv8n + DSCf	83.9	74.4	83.1	**4.4**	**2.59**
YOLOv8n + DSCf + CA	85.6	75.2	84.1	4.5	2.60
YOLOv8n + DSCf + CA + SIOU	86.3	74.5	84.3	4.5	2.60
TBF-YOLOv8n	**87.5**	74.4	**85.0**	4.5	2.61

**Table 7:** Results of loss function ablation experiments.

Model	P/%	R/%	${mAP}_{50}/\%$	mAP_50:95_/%
YOLOv8n(CIOU)	83.8	**75.6**	83.9	61.5
YOLOv8n_SIOU	**84.4**	75.0	**84.0**	**61.6**

**Table 8:** Visualization of test results.

Model	True	False	Miss	FLOPs/G	Params/M	P/%
YOLOv7-tiny	8	2	1	13.2	6.01	68.6
YOLOv8n	9	0	0	8.1	3.01	68.0
YOLOv9-tiny	7	1	2	10.7	2.62	68.3
YOLOv10n	9	1	0	8.2	2.70	90.5
YOLO11n	9	1	0	6.3	2.58	73.1
YOLOv5_tea [25]	9	1	0	22.7	9.20	85.1
YOLOv8_tea [39]	8	0	1	9.40	4.61	81.1
TBF-YOLOv8n	9	0	0	**4.5**	2.61	78.6

## Data Availability

The data in this study are available upon request from the corresponding author.
